# Biodegradable Microspheres for Transarterial Chemoembolization in Malignant Liver Disease

**DOI:** 10.3390/medicina60040678

**Published:** 2024-04-22

**Authors:** Ornella Moschovaki-Zeiger, Nikolaos-Achilleas Arkoudis, Athanasios Giannakis, Stavros Grigoriadis, Fotis Anagnostopoulos, Stavros Spiliopoulos

**Affiliations:** 1 2nd Department of Radiology, School of Medicine, “Attikon” University General Hospital, National and Kapodistrian University of Athens, GR-124 62 Chaidari, Greece; m.z.ornella@gmail.com (O.M.-Z.); narkoudis@med.uoa.gr (N.-A.A.); a-giannakis@hotmail.com (A.G.); grigoriadis_stavros@hotmail.com (S.G.); anagnostopoulosfotis@gmail.com (F.A.); 2 Research Unit of Radiology and Medical Imaging, 2nd Department of Radiology, Medical School, National and Kapodistrian University of Athens, GR-115 28 Athens, Greece

**Keywords:** transarterial chemoembolization (TACE), degradable microspheres (DMS), locoregional therapy, liver malignancies

## Abstract

Transarterial chemoembolization (TACE) has revolutionized the treatment landscape for malignant liver disease, offering localized therapy with reduced systemic toxicity. This manuscript delves into the use of degradable microspheres (DMS) in TACE, exploring its potential advantages and clinical applications. DMS-TACE emerges as a promising strategy, offering temporary vessel occlusion and optimized drug delivery. The manuscript reviews the existing literature on DMS-TACE, emphasizing its tolerability, toxicity, and efficacy. Notably, DMS-TACE demonstrates versatility in patient selection, being suitable for both intermediate and advanced stages. The unique properties of DMS provide advantages over traditional embolic agents. The manuscript discusses the DMS-TACE procedure, adverse events, and tumor response rates in HCC, ICC, and metastases.

## 1. Introduction

The treatment options for malignant liver diseases have evolved significantly with the introduction of transarterial chemoembolization (TACE), a therapeutic method that combines chemotherapeutic agents with embolic materials consisting of Lipiodol or drug-eluting beads (DEB), or more recently, degradable microspheres (DMS). The purpose of TACE is to create a significant drug concentration gradient within the tumor, minimizing systemic concentrations and thereby limiting systemic side effects, while concurrently enhancing local antitumor efficacy [1]. This manuscript investigates the use of DMS, an innovative degradable embolic/carrier material, for the treatment of malignant liver diseases, focusing on its potential advantages and the limited existing data on its tolerability, toxicity, and effectiveness [2].

Hepatocellular carcinoma (HCC) and intrahepatic cholangiocarcinoma (ICC) constitute the two main primary liver malignancies, contributing significantly to global cancer-related morbidity and mortality [3,4]. While TACE, particularly instances using Lipiodol, remains a standard therapeutic modality for intermediate-stage HCC [5], the options for advanced-stage cases and other hepatic malignancies are limited. DMS-TACE emerges as a promising strategy, offering a regulated temporary vessel occlusion and optimized binding capacities for chemotherapeutic agents. Unlike conventional TACE (cTACE) techniques with prolonged washouts [6], recently available data demonstrate that DMS-TACE allows for temporary occlusion, minimizing post-embolization syndrome and providing sufficient flexibility for repeated treatments [7].

Despite its potential, there is a notable absence of comprehensive data on DMS-TACE’s tolerability, toxicity, and efficacy. This manuscript aims to bridge this knowledge gap by reviewing existing studies, emphasizing the benefits of temporary occlusion, shorter ischemia times, and the ability to reperform treatment, especially in cases of bilobar extensive disease or when selective treatment proves challenging. Furthermore, the manuscript explores recent applications of DMS-TACE in advanced HCC, ICC, and liver metastases, offering insights into its evolving role in the rapidly advancing landscape of interventional oncology.

## 2. Indications and Patient Selection

TACE employing DMS emerges as a versatile therapeutic option, supported by its accreditation for the treatment of primary and secondary liver tumors, underscoring its wide-ranging clinical utility. This approach is advocated in instances necessitating the occlusion of tumor feeding arteries, facilitating the repetition of procedures as required. Notably, DMS-TACE exhibits efficacy even in patients diagnosed at advanced stages where curative interventions such as ablation, resection, or transplantation are no longer feasible.

Recent literature highlights the use of degradable embolic materials in selected patients with advanced HCC [8,9,10], as well as in ICC [11] and metastases from colorectal cancer (CRLM) [12]. Furthermore, DMS-TACE protocols are offered to patients as supportive palliative care, particularly those suffering from advanced and aggressive disease. Particularly in cirrhotic patients with unresectable HCC, DMS-TACE has been extensively employed as a therapeutic option [13]. A major advantage is its utility in patients with elevated bilirubin levels exceeding 3 mg/dl and those with portal vein thrombosis, where alternative treatment options may be contraindicated [8,14]. 

DMS-TACE is employed as both first-line [10,15] and second-line treatment for HCC, particularly after Sorafenib cessation [8], showcasing its adaptability in different clinical scenarios. Additionally, it demonstrated efficacy as a bridging therapy in preventing dropout from the waiting list for patients with HCC Child-Pugh stage B, with outcomes comparable to those observed with other locoregional treatments [16]. In cases of advanced-stage HCC disease necessitating multiple sessions of lobar treatments, DMS-TACE emerges as an ideal therapeutic option. Repetitive DMS-TACE emerges as a viable treatment option for all HCC patients with a high or diffuse tumor burden and those not suitable for or failing other curative or palliative treatment options [14].

Overall, DMS-TACE represents a versatile therapeutic approach for patients with bilobar extensive disease or when selective treatment modalities cannot be performed, making it a valuable addition to the armamentarium of treatments for liver malignancies [14].

## 3. Product Profile and Pathophysiology

DMS-TACE represents a significant advancement in the management of malignant liver lesions. This technology addresses several critical aspects of the embolization process. Firstly, it reduces in-lesion drug wash-out, functioning as an embolic agent. Additionally, the short-term ischemia induced by DMS-TACE results in a less aggressive temporary embolic effect, which not only prevents liver function deterioration but also decreases growth factor proliferation that typically enters the bloodstream after a few hours of hypoxia [14,17].

Within the context of the TACE method, the embolic agent is an essential component since it is responsible for transporting the chemotherapeutic agent to the target area and creating blood vessel obstruction [18,19]. Moreover, ischemia induced by embolization can lead to tumor necrosis [20] and alter cellular signaling pathways, possibly facilitating chemotherapeutic agent uptake and cytotoxic action [21]. They function as carriers without chemically adhering to the drugs and are fully absorbed within about 2 h [8], compared with Lipiodol, which takes 5–12 weeks to wash out, and DEBs, which cause permanent vascular occlusion [2,5,22,23].

In comparison to traditional embolic agents like Lipiodol and permanent occlusive drug-eluting beads, DMS microspheres have a number of characteristics that provide them with an advantage. DMS-TACE avoids the systemic vascular endothelial growth factor (VEGF) response, which may facilitate tumor growth and metastatic seeding [24,25], associated with cTACE and DEB-TACE [26], and spares the smaller vessels, reducing the risk of ischemia-reperfusion injury [27]. The unique anatomic properties of the liver, with its dual blood supply, necessitate careful consideration during embolization procedures. Transient occlusion of tumor feeding arteries using DMS helps avoid VEGF overexpression and provides temporary vessel occlusion, with a half-life of approximately 35–30 min [7,8]. DMS combine transient vascular occlusion with optimized binding capacities for chemotherapeutic agents, leading to increased intratumoral concentrations [19,28]. Temporary occlusion reduces the activation of hypoxia-inducible factors and the secretion of VEGF, making DMS-TACE a powerful therapeutic strategy for controlling these lesions. Despite being available for decades [29], DMS have recently emerged as a viable alternative to conventional embolic agents that may be combined with a variety of chemotherapy agents [8]. DMS-TACE offers several benefits, including shorter ischemia time and the ability to reperform treatment on the same feeding vessels [8,9,10,13,30]. Commercially available degradable microparticles for DMS-TACE include the following:

- Starch microspheres (EmboCept DMS 35/50, Embocept^®^ (Sirtex Medical Inc., Woburn, MA, USA)): a degradable short-term embolic agent composed of starch microspheres with an average diameter of 50 micrometers. The microspheres have a half-life of about 35–50 min and are broken down by serum alpha-amylases in the blood [8,12,31]. Most currently available clinical data on DMS-TACE are based on this product.
- Gelatin microspheres (Gel-Bead, Teleflex, Morrisville, NC, USA): available in various diameters (100–300; 300–500; 500–700; and 7000–1000 μm) and presenting a degradation period between 4 and 12 weeks.
- Polylactic-co-glycolic acid (PLGA) Microspheres (Occlusin^®^ 500 Artificial Embolization Device, IMBiotechnologies, Edmonton, AL, Canada): a hydrophobic, degradable polymer available in 150–210 μm diameter, but presenting a long-term complete degradation time (6 to 12 months). The product has obtained FDA approval for use in hypervascularized tumors with no surgical options [32].

Numerous investigations have thoroughly examined the in vitro functionality of biodegradable microspheres, with a significant emphasis on assessing their degradation rates [32]. Nevertheless, disparities between in vivo and in vitro degradation observations highlight the imperative for additional comprehensive research studies. The ideal degradation timeframe for embolic microspheres remains indistinct, potentially varying across different experimental animal models, human subjects, and even among distinct organs. These observations emphasize the critical necessity for meticulous preclinical studies aimed at clarifying the pharmacokinetic characteristics and safety considerations associated with biodegradable microspheres.

## 4. DMS-TACE Procedure 

The DMS-TACE procedure conventionally involves the preparation of the drug immediately prior to administration (as per product IFU). This solution is subsequently combined with adjunctive non-ionic contrast medium and delivered via a coaxially positioned microcatheter to mitigate reflux [33,34,35]. Dosage adjustments are not contingent upon serum bilirubin levels or patient surface area, as these factors are deemed to be independent of dosage determination [13]. The therapeutic approach encompasses the administration of the anticipated total chemotherapeutic drug dosage, followed by the delivery of the DMS as an unloaded temporary embolic agent to induce transient cessation of flow within the treated artery. Supplementary embolic material such as DMS or gel-foam may be required based on vessel selectivity and diameter, with the ultimate objective of achieving stasis within the tumor feeding vessels to facilitate adequate drug absorption in the target lesion. 

The primary objective of the technique is to optimize drug delivery to the liver while concurrently minimizing the ischemic duration and mitigating the risk of post-embolization syndrome, given the half-life of DMS [8]. In cases involving bilobar tumor presence, treatment is sequenced to avert compromise to liver function, initially prioritizing the treatment of the lobe with greater tumor burden, followed by intervention on the contralateral lobe after a fourteen-day interval, with subsequent treatment cycles recurring every two weeks thereafter [35]. 

The scheduling of DMS-TACE procedures is typically governed by staging imaging (MRI and/or CT) and subsequent restaging, with intervals between interventions typically falling within a range of 2–6 weeks [12,14,36]. Standard vascular access, typically through the femoral or radial artery, is established in all DMS-TACE procedures, with catheterization of the hepatic artery conducted in a selective or super-selective manner based on considerations including tumor burden and hepatic vascular status. Various chemotherapeutic agents, including mitomycin, gemcitabine, cisplatin, carboplatin, and doxorubicin, are frequently employed in diverse combinations [37].

Unlike cTACE, DEB-TACE, and SIRT procedures, DMS-TACE necessitates repetitive interventions until the tumor’s control becomes unattainable or other factors necessitate treatment cessation. It is recommended that a minimum of three treatments, with the possibility of up to six sessions, be attempted before drawing conclusions regarding its efficacy and potentially dismissing this technique as an effective treatment option [14].

**Figure 1:**
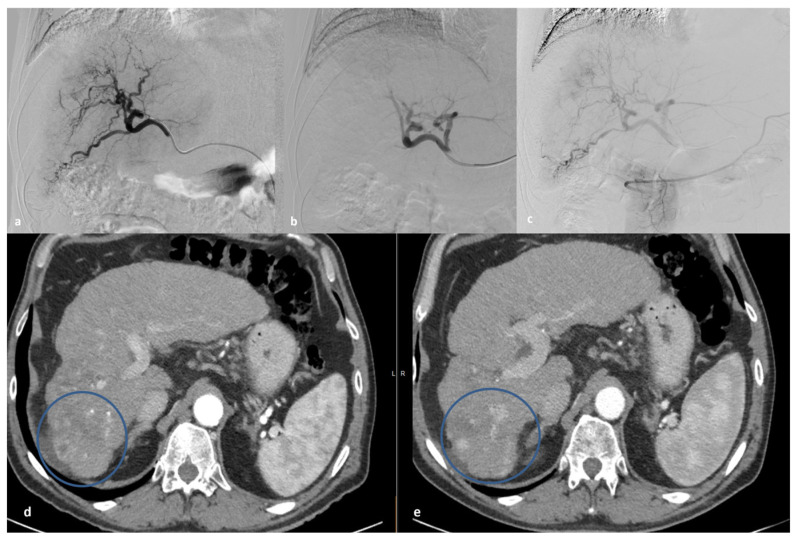
A 63-year-old cirrhotic patient with multifocal right lobe HCC who underwent 3 sessions of doxorubicin DMS-TACE. (**a**) Digital subtraction angiography (DSA) from the right hepatic artery depicting the arterial vasculature and the HCC hypervascular lesions of the right hepatic lobe. (**b**) Final DSA following the 1st session of whole-lobe TACE using 100 mg of doxorubicin and degradable starch microspheres, demonstrating complete devascularization of the right hepatic lobe. (**c**) Initial DSA prior to the 2nd DMS-TACE session (1 month after the 1st session), demonstrating complete viability of the right-lobe arterial supply, including the feeding vessels of the target HCC lesions. (**d**) A 1-year follow-up CT (arterial phase) demonstrating sustained complete response of the treated lesions (circle), compared to preprocedural imaging (**e**), while aFP levels remain decreased, at normal values.

Combining radiofrequency ablation (RFA) with DMS-TACE has been described as a promising approach for the treatment of CRLM [38], and more recently, it has been explored for other non-HCC primary or metastatic malignant liver lesions [39]. This combined therapy offers the advantages of both modalities, while studies have demonstrated encouraging results, with high rates of tumor control and the possibility of managing local tumor progression with repeated sessions, as well as prolonged overall survival [39]. Moreover, the synergistic effects of ablation and TACE may facilitate complete tumor eradication and reduce the likelihood of tumor recurrence, particularly in cases where lesions are larger or located in challenging anatomical locations [40,41]. Additionally, the degradable nature of DMS allows for repeated treatment if necessary, enabling the adaptation of therapeutic approaches based on tumor response and disease progression [39], exemplified in the case illustrated in Figure 1.

## 5. DMS-TACE Treatment: Adverse Events—Results

Based on various studies, an array of adverse events (AEs) associated with DMS-TACE procedures have been reported. Minici et al. noted that 29.6% of 54 patients experienced postprocedural clinical complications following TACE, primarily consisting of grade 1 complications (according to the CIRSE Classification System), such as pain, post-embolization syndrome, transient nausea, and vomiting, with an incidence of 25.9%. Notably, only two cases (3.7%) were classified as grade 3, both involving non-surgical cholecystitis that required no further intervention [16]. Similarly, Schicho et al. reported that the majority (32%) of treatment-emergent adverse events (TEAEs) occurred within the 24 h following DMS-TACE procedures; these primarily included nausea, vomiting, and epigastric pain and were mostly related to the combination of mitomycin, gemcitabine, and cisplatine and gemcitabine alone. Notably, one (4%) treatment-emergent severe adverse event (TESAE) that occurred in-between treatments, an event involving thrombocytopenia and intracranial subdural hemorrhage, led to the discontinuation of the treatments [37]. In another study by Schicho et al. on DMS-TACE for intermediate stage HCC, two patients (4%) had to discontinue the TACE session due to an immediate adverse event of allergic reaction, although 48% of the patients included in the study experienced no immediate adverse event or severe adverse event overall. Between treatments, epigastric pain and nausea/vomiting were also reported, along with diarrhea, transient neutropenia, thrombocytopenia, and one case of gastric ulcer [10]. In a study the following year, Schicho et al. observed that in a total of 77 DMS-TACE procedures, no immediate or severe AE was recorded, indicating an overall favorable safety profile for DMS-TACE [12]. An additional observation by Orlacchio et al. was of the transient increases in serum aspartate aminotransferase/alanine transaminase (AST/ALT) and gamma-glutamyl-transpeptidase (GGT) concentrations in most patients within 24 h post-procedure [42], an observation that is generally noted in TACE [43]. Haubold et al. documented complications during or in-between 134 DMS-TACE interventions, with 11% classified as CIRSE grade 1, 9% as CIRSE grade 2, and 2% as CIRSE grade 3, indicating a spectrum of severity in complications associated with DMS-TACE [30]. In 21 patients that underwent 64 DMS-TACE sessions for intrahepatic cholangiocarcinoma, five adverse events were reported, out of which two were lethal, including one case of severe pyogenic liver abscess related to the intervention, but most probably due to the lack of compliance of the patient, and another sudden event, involving cardiac arrest, most probably irrelevant to the intervention [11]. Another study involving 137 patients who underwent a total of 267 DMS-TACE sessions for unresectable HCC (stages A, B, and C) reported one death, attributed to extensive hepatic artery occlusion and subsequent liver failure. Additionally, four major complications, accounting for 6.8% of cases, were documented, including one case of cholecystitis, two cases of hepatic abscess, and one case of massive portal vein thrombosis and gastroesophageal variceal bleeding [13]. In a smaller study conducted on 37 patients with unresectable HCC, comprising both uninodular and multinodular diseases, a total of 177 procedures were performed with doxorubicin or epirubicin, which resulted in predominantly CIRSE grade-1 adverse events. These included pain, transient nausea, vomiting, and post-embolization syndrome. Only one treatment-related CIRSE grade-3 event was observed, involving a duodenal ulcer with microperforation [9]. The adverse events of studies investigating DMS-TACE are analytically reported in Table 1.

## 6. DMS-TACE Efficacy and Tumor Response—Results

### 6.1. HCC

The majority of HCCs are diagnosed at stages where curative resection, ablation, or transplantation are no longer viable treatment options, particularly for intermediate- or advanced-stage HCC patients [22,44]. TACE is indicated as the first-line treatment for patients with intermediate-stage HCC, or stage B disease (as classified by the Barcelona Clinic Liver Cancer Staging System (BCLC)), as stated by the recommendations provided by the European Association for the Study of the Liver (EASL) guidelines [22,45,46], while it is also widely used among locoregional therapies for downstaging [47,48,49]. TACE is also indicated for unresectable, single, or multinodular HCC in patients with preserved liver function and no evidence of vascular invasion [50]. Several studies have reported on the efficacy and tumor response of the DMS-TACE procedure. Kirchhof et al. conducted a study involving 47 HCC patients treated with DMS and Lipiodol mixed with doxorubicin and cisplatin, revealing a median survival rate of 26 months and a 1-year survival rate of 75% [51]. Another research study, by Gross et al., on patients with locally more extensive HCC disease revealed an objective response rate (complete or partial) of 49% and a disease control rate (complete/partial response or stable disease) of 83%, according to mRECIST. Most patients (94%) achieved the best treatment response after one cycle, with progression observed in 45% of patients at some point. Notably, 35% of patients with progressive disease responded to DMS-TACE upon restarting the treatment. However, BCLC stage C and BCLC stage D patients did not show a survival benefit despite promising tumor response rates [9]. Haubold et al. evaluated the tumor response following three treatments of DMS-TACE and found the overall rate of complete response (CR) to be 14.3%, partial response (PR) to be 25%, stable disease (SD) to be 39.3%, and progressive disease (PD) to be 21.4%, according to the mRECIST criteria. Their study demonstrated an overall good level of median survival of 682 days, with survival outcomes significantly dependent on the BCLC stage [30]. Moreover, repetitive DMS-TACE has demonstrated the preservation of liver function over time, even in patients whose entire liver is treated [14]. 

The efficacy of DMS-TACE in terms of tumor response is noteworthy, with high response rates observed, even after only one treatment. Moreover, significant differences in overall survival and time to progression have been reported in patients with more than 50% necrosis after the first procedure [13]. Studies have shown that the addition of DMS to cTACE resulted in a significant benefit in tumor response compared to TACE with Lipiodol, although no difference in survival time was observed [52]. Yamazaki et al. compared the efficacy of Lipiodol with DMS versus Lipiodol alone, revealing tumor response rates of 80% and 40%, respectively, with significantly longer progression-free intervals observed in the former group [15]. Additionally, TACE plus DMS has been associated with a lower percentage of side effects and increased patient tolerance, compared to TACE with Lipiodol alone [2]. In a retrospective study that compared the efficacy of DMS-TACE using microspheres with an average diameter of 50 ± 7 µm to DEB-TACE using beads with a size range of 300–500 μm in 54 patients, local recurrence was observed in 28.0% of patients, and mortality occurred in 42.6%, while there was no significant disparity between DMS and DEB concerning local recurrence or mortality rates. Interestingly, a complete response was achieved in 14.8% of the patients, with a notably higher rate in the DMS-TACE group compared to the DEB-TACE group. Furthermore, there was no substantial difference between the partial responses observed in the two groups [53].

Minici et al. conducted a study on 54 early-stage HCC patients with Child–Pugh stage B undergoing DMS-TACE as bridging therapy for liver transplantation, and they found an overall survival rate of approximately 96% at 6 months and 92% at 12 months, with 33% of patients successfully undergoing liver transplantation [16]. Furthermore, Schicho et al. analyzed 179 DMS-TACE procedures in 50 patients, observing an objective response rate of 44% and a disease control rate of 70%, indicating the effectiveness of the technique [10]. Orlacchio et al. reported on tumor response in 24 cirrhotic HCC patients, noting complete response rates of 20.8%, 23.5%, and 41.6% after the first, second, and third procedures, respectively. At the end of each treatment, all patients experienced at least a partial response, and there were no significant differences observed between mono- or bilobar disease in patients achieving a complete response [42].

Overall, DMS-TACE demonstrates promising efficacy and tumor response rates, making it a valuable option for patients with HCC, particularly those not amenable to curative treatments.

### 6.2. ICC

Research has demonstrated the efficacy of TACE treatments, such as DMS-TACE, in improving survival outcomes for ICC compared to systemic therapy [54]. However, data on DMS-TACE as a management option for ICC remain limited due to the rarity of the tumor and the absence of standardized treatment regimes. Nonetheless, an analysis involving 18 DMS-TACE procedures revealed somewhat promising therapeutic outcomes, with PR observed in 12% of the cases, while SD was recorded in 32%. DP was registered at 4%, while 28% of the cases were lost on follow-up. Additionally, DMS-TACE exhibited a disease control rate of 44% in seven patients, underscoring its potential effectiveness [37]. Furthermore, another research study revealed notable imaging responses according to RECIST criteria, with complete remission observed in 11.1% of patients, partial response in 50%, and stable disease in 38.9% of patients. This yielded an objective response rate of 61.1% and a disease control rate of 100% [11]. These findings highlight the favorable therapeutic efficacy and tumor response associated with DMS-TACE in the management of ICC.

### 6.3. Metastases

The therapeutic efficacy of the DMS-TACE procedure, as evaluated by the mRECIST criteria, demonstrated varying degrees of response in patients with hepatic metastases. DMS-TACE has shown promising disease control rates, with 44% of patients demonstrating disease control in a study by Schicho et al. on patients with CRLM. Among the 77 treatments in the study, a complete response was not observed, while a partial response was noted in 17 cases, stable disease in 33 cases, and progressive disease in 6 cases. Overall, an objective response was achieved in 40.0% of cases and disease control in 64.9% of procedures [12]. Studies comparing DMS-TACE with cTACE have shown similar tumor response rates, with DMS-TACE demonstrating significant tumor volume reduction and comparable median survival rates. Additionally, survival analysis revealed no significant difference in 1-year survival rates between cTACE and DMS-TACE in patients with CRLM [55]. Retrospective analyses have reported a median overall survival of 13.8 months in CRLM patients and a mean survival of 15.5 months in HCC patients following DMS-TACE treatment, indicating the potential efficacy of DMS-TACE [2,56]. 

Outcomes of studies investigating DMS-TACE are analytically reported in Table 2.

## 7. DMS-TACE versus Others

Limited randomized trials have explored the comparative efficacy of degradable starch microsphere transarterial chemoembolization (DSM-TACE) versus cTACE for treating malignant liver lesions. In a prospective study conducted by Vogl et al., involving 31 patients with CRLM, no statistically significant disparity in median survival was observed between the two groups. However, the DSM-TACE cohort exhibited a substantial decrease in tumor volume compared to cTACE, with stable disease reported in 56% versus 15%, respectively, and progressive disease in 22% versus 62%, respectively [55]. Conversely, a study by Niessen et al., comprising 69 patients with intermediate-stage HCC, found comparable objective response rates and rates of stable disease between the DSM-TACE and cTACE groups, with no significant variance in mean survival or complications [2]. No increased efficacy of DSM-TACE over TACE alone was reported by Kirchhoff et al. in advanced unresectable HCC, though DSM-TACE exhibited a lower overall incidence of complications compared to cTACE [51]. These findings underscore the need for further large-scale trials to discern the optimal embolizing agents that maximize therapeutic benefits while minimizing adverse effects, thus improving overall survival rates. 

## 8. Discussion

With the introduction of drug eluting beads, an attractive alternative in TACE procedures has been offered, providing safe and efficient treatment options [18,57,58]. However, DEB-TACE may lead to prolonged ischemia and VEGF stimulation, potentially contributing to tumor recurrence [26,59,60]. The efficacy and safety of DMS-TACE in treating malignant liver lesions are a subject of growing interest, as transient occlusion of tumor feeding vessels allows for repeated treatments within a short period, with minimal damage to non-tumoral hepatic tissue [7,29]. Data on the safety and efficacy of DMS-TACE, although limited, are promising, showing favorable results compared to cTACE and DEB-TACE [2,5,10,42,61]. One of the notable advantages is its applicability in patients with specific clinical conditions, such as a bilirubin level greater than 3 mg/dl and portal vein thrombosis, expanding the treatment options for these individuals [8]. However, although DMS-TACE presents a promising option, there is still a lack of high-quality data regarding the optimal chemotherapeutic and embolization treatment protocols in DMS-TACE [10], and its clinical usefulness remains under investigation [2,7,15,34]. 

This review explores the implications and insights gleaned from the extensive literature on DMS-TACE in the management of malignant liver lesions. Our synthesis of the existing body of evidence underscores the multifaceted nature of DMS-TACE as a therapeutic modality and its potential impact on clinical practice. Further large-scale, prospective, and comparative studies are necessary to address these gaps and optimize the clinical outcomes of DMS-TACE in the management of malignant liver lesions.

In the context of preclinical evaluation of DMS for use in TACE for malignant liver lesions, it is essential to address the need for specific controls tailored to assess their safety and efficacy. There exists a notable gap in our current understanding of the characteristics and performance of degradable microspheres, leading to uncertainty regarding their optimal properties. The unique degradability of these microspheres introduces novel risks, with migration being a primary concern. As these microspheres degrade, they may undergo size reduction, potentially leading to unintended migration beyond the level of the lesion. Investigating microsphere migration poses significant challenges due to the variabilities in vascular patterns and blood flow among different species and disease states. Therefore, further preclinical evaluation protocols must be established to comprehensively assess the migration behavior and associated risks of DMS in TACE applications for malignant liver lesions [32].

## 9. Future Directions

The future directions for DMS-TACE in the management of liver malignancy encompass a wide array of potential advancements aimed at enhancing its efficacy and safety profile. One potential path is the refinement of DMS-TACE techniques to enhance their efficacy and safety profile in the long term and with larger patient-cohort studies. This may involve optimizing the selection and combination of chemotherapeutic agents delivered with DMS to maximize therapeutic efficacy while minimizing adverse events. Additionally, efforts are underway to perfect the embolization process, aimimg to achieve more efficient and durable tumor occlusion. Concurrently, researchers are investigating novel degradable materials such as chitosan, PLGA-PEG-PLGA, and hydroxyethyl acrylate (HEA) in pre-clinical studies, with anticipation of future human trials [32]. The integration of DMS-TACE with other treatment modalities, including immunotherapy and targeted molecular therapies, represents another frontier of exploration. By combining DMS-TACE with these emerging therapies, researchers aim to evaluate the synergistic effects and improve overall treatment outcomes. Furthermore, advancements in imaging technology hold promise for facilitating the precise delivery of DMS to tumor sites, thereby enhancing treatment precision and efficacy. Ultimately, the continued refinement and innovation in DMS-TACE technology, techniques, and strategies hold great potential for improving the therapeutic outcomes for patients with liver malignancy. However, large, multi-center, prospective randomized trials designed to provide comparable data addressing DMS and the treatments currently suggested by international guidelines for conventional or drug-eluting microparticle TACE are imperative to support the use of DMS in everyday clinical practice.

## 10. Conclusions

DMS-TACE represents a promising and versatile therapeutic approach for the treatment of malignant liver disease, including HCC, ICC, and liver metastases. By combining the benefits of temporary vessel occlusion with optimized drug delivery, DMS-TACE offers advantages such as shorter ischemia time, minimized post-embolization syndrome, and the ability to repeat treatments as needed. However, care must be taken in achieving optimal preparation and infusion technique, as suggested for each product, in order to achieve the maximal antitumoral effect. Despite the limited existing data, DMS-TACE has demonstrated favorable safety and efficacy profiles, with promising tumor response rates and disease control outcomes. Its applicability in patients with specific clinical conditions further expands the treatment options for individuals who may not be candidates for other modalities. However, further comparative studies are needed to optimize treatment protocols and determine long-term clinical outcomes. Overall, DMS-TACE represents a valuable addition to the armamentarium of treatments for liver malignancies, holding great promise in the rapidly advancing landscape of interventional oncology.

## Figures and Tables

**Table 1:** Adverse events of DMS-TACE in the current literature.

Study	Year	Number of Patients	Minor Adverse Events/Toxicity	Major Adverse Events/Toxicity
Minici et al. [16]	2021	54	Pain, post-embolization syndrome, transient nausea, vomiting: 25.9%	Cholecystitis: 3.7%
Schicho et al. [37]	2017	7	Nausea, vomiting, epigastric pain: 32%	Thrombocytopenia: 4%
Schicho et al. [10]	2017	50	Epigastric pain, nausea, vomiting, diarrhea, transient neutropenia, thrombocytopenia: 48%	Allergic reaction: 4%
Haubold et al. [30]	2020	28	CIRSE Grade 1: 11%; CIRSE Grade 2: 9%	CIRSE Grade 3: 2%
Goerg et al. [11]	2019	21	Asymptomatic peribiliary necrosis: 9.5%	Hepatobiliary abscess: 4.8%; Death: 9.5%
Orlacchio et al. [13]	2020	137	Post embolization syndrome: 73.7%	Cholecystitis, hepatic abscess, massive portal vein thrombosis: 68%; Death: 1.4%
Gross et al. [9]	2020	37	Pain: 23%; Nausea: 11%; Vomiting: 3%	Duodenal ulcer: 0.4%

**Table 2:** Outcomes of DMS-TACE in the current literature.

Study	Year	Malignancy	Outcomes
Kirchhof et al. [51]	2006	HCC	PR: 26%; SD: 41%; PD: 33%
Gross et al. [9]	2020	HCC	Objective response rate: 49%; Disease control rate: 83%
Haubold et al. [30]	2020	HCC	CR: 14.3%; PR: 25%; SD: 39.3%; PD: 21.4%
Yamazaki et al. [15]	2011	HCC	Tumor response rate: 80%
Minici et al. [16]	2021	HCC	Overall survival rate: 96% at 6 months, 92% at 12 months
Schicho et al. [10]	2017	HCC	Objective response rate: 44%; Disease control rate: 70%
Orlacchio et al. [42]	2018	HCC	Complete response rates: 20.8%, 23.5%, and 41.6% after the first, second, and third procedures, respectively
Schico et al. [37]	2017	ICC	PR: 12%; SD: 32%
Goerg et al. [11]	2019	ICC	Complete remission: 11.1%; PR: 50%; SD: 38.9%
Schicho et al. [12]	2018	CRLM	Disease control rate: 44%; Objective response: 40%
